# One-Year Changes in Collagen Type in Semitendinosus Tendons: A Case Study Using Tissues Obtained From a Growing Patient

**DOI:** 10.7759/cureus.67564

**Published:** 2024-08-23

**Authors:** Yushin Mizuno, Yasushi Takata, Kazuaki Yoshioka, Satoru Demura, Junsuke Nakase

**Affiliations:** 1 Department of Orthopedic Surgery, Graduate School of Medical Sciences, Kanazawa University, Kanazawa, JPN; 2 Section of Rehabilitation, Kanazawa University Hospital, Kanazawa, JPN; 3 Department of Physiology, Graduate School of Medical Sciences, Kanazawa University, Kanazawa, JPN

**Keywords:** anterior cruciate ligament (acl) reconstruction, collagen type composition, traction force, semitendinosus tendon, bone growth, age-related changes

## Abstract

Recently, there have been concerns about the high postoperative re-injury rate associated with the use of the semitendinosus tendon (ST) as an autograft for anterior cruciate ligament reconstruction in adolescent patients before the closure of the epiphyseal line. Our previous studies showed that this high re-injury is related to the histological and mechanical immaturity of ST in adolescent patients. Moreover, the overall structure of collagen fibers is strengthened with the application of traction force to tendon tissue. Therefore, it is assumed that, in vivo, bone growth and increased height increase the traction force applied to tendon tissue and the percentage of type I collagen, which has a remarkable physical strength. The present study aimed to investigate the changes in the content of ST's type I collagen in an adolescent patient over one year. The patient was an 11-year-old male with bilateral patellar dislocations. The orthopedic surgeon performed medial patellofemoral ligament reconstruction on the left knee using an ST graft, followed by a similar procedure on the right knee one year later. ST tissue that would have been discarded during each procedure was harvested and used. The bone of the patient's legs grew approximately 8 cm during the one-year period. The obtained tissues were immunostained and microscopically observed to evaluate the area content of type I and III collagen. The area content of type I collagen in STs collected from the patient was 66%. The area content of type I collagen increased rapidly to 95% one year later. A comparison of the two STs obtained from the patient in the first half of their 10^th^ year showed that the type I collagen content of the STs increased rapidly over one year. This fact may provide a preliminary insight into the prevention of re-injury when selecting the autograft for anterior cruciate ligament (ACL) reconstruction in adolescent patients.

## Introduction

Anterior cruciate ligament (ACL) injuries are the most common sports knee injuries, and reconstruction using the patient's own tendon is the most conventional treatment [1]. Semitendinosus tendons (ST) are widely used as autografts worldwide because of their ease of harvesting and minimal post-harvesting problems [2]. Recently, a high postoperative re-injury rate after transplantation of STs in adolescent patients with an open epiphyseal line has been reported [3], attracting attention from clinicians, especially orthopedic surgeons. Factors responsible for this high re-injury rate include the high level of sports to which the adolescent patient returns and good pain management at one year postoperatively [4]. In addition to these factors, a possible cause is the immaturity of the autograft, which is in the process of growth, as is the body of the adolescent patient. The collagen fibril diameter of the ST of patients with an open epiphyseal line is significantly smaller than that of adults [5]. Furthermore, STs contain a lower amount of type I collagen, which is associated with physical strength, than do patellar and quadriceps tendons that can be utilized as autografts [6].

The main component of tendons is type Ⅰ collagen, which is rod-shaped and stiff and possesses a high mechanical strength [7]. Type Ⅲ collagen, also present in tendons, is more flexible than type Ⅰ collagen [8]. This difference in physical strength may be related to the longevity of STs harvested as autografts. In addition, traction forces on tendon tissue are believed to structurally strengthen the entire collagen fiber [9]. The traction on tendon tissue in vivo can be increased through bone extension, that is, body growth. Thus, the collagen type associated with the physical strength of tendon tissue may change as the body grows and the tendon undergoes traction. However, no study has examined the changes in ST collagen type associated with body growth using tissue of biological origin.

We had the rare opportunity to collect STs from the same patient during the growth period, with a one-year interval. The study aimed to investigate changes in collagen type content of STs over one year in the same adolescent patient. We hypothesized that the percentage of type I collagen in STs increases with the patient’s height over one year.

## Case presentation

This study was conducted with the approval of the Ethics Review Committee of our university. The patient and their parents received oral and written information about the study and provided their consent. The level of evidence of this study is V (case report). 

Patient information

**Figure 1:**
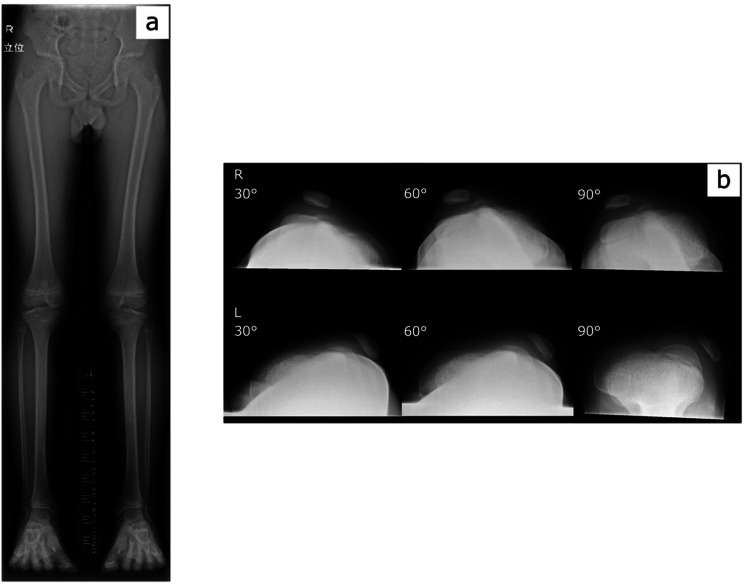
Standing radiograph and skyline images of the patella at 11 years of age Full-length radiographs of the standing lower limb (a) and skyline images of the patella (b) obtained before surgery on the left knee at 11 years of age.

The patient was an 11-year-old male with a history of left patellar dislocation with knee flexion and extension for several years. The patient visited our hospital due to a recent increase in the frequency of dislocation, which became painful. Clinical findings showed that the left patella was easily dislocated at 15˚ of knee joint flexion and the right patella was dislocated at 90˚ of knee joint flexion. Radiographs of the knee in the standing position (Figure 1a) and skyline images of the patella (Figure 1b) were obtained. Based on these findings, the orthopedic surgeon diagnosed the patient with bilateral habitual patellar luxation and performed surgery on the left knee. On initial admission, the patient's height and weight were 139.2 cm and 29.3 kg, respectively. The spina malleolar distance (SMD) calculated from standing radiographs was 73.1 cm on the right and 74.7 cm on the left.

**Figure 2:**
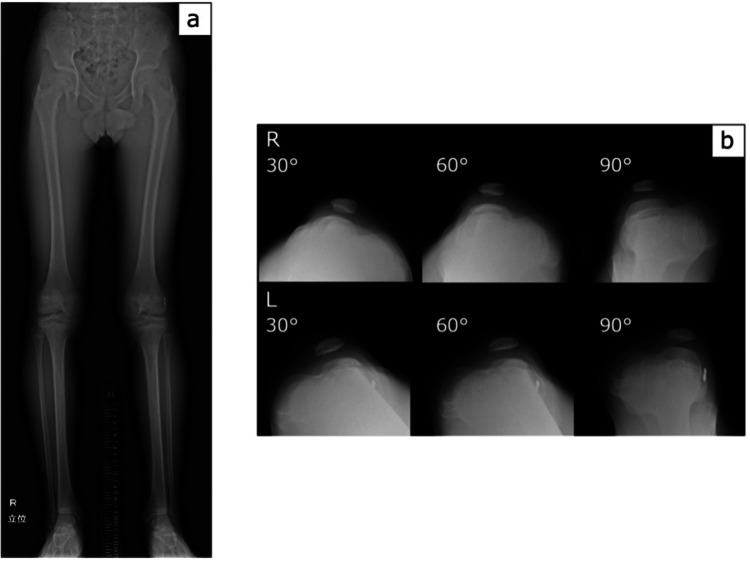
Standing radiograph and skyline images of the patella at 12 years of age Full-length radiographs of the standing lower limb (a) and skyline images of the patella (b) obtained before surgery on the right knee at 12 years of age.

Exactly one year after surgery on the left knee, surgery was performed on the right knee. In addition, radiographs of the knee in the standing position (Figure 2a) and skyline images of the bilateral patella (Figure 2b) were obtained before the second surgery. The patient’s height and weight were 147.9 cm and 35.5 kg, respectively; the patient’s SMD was 81.4 cm on the right and 81.6 cm on the left, indicating approximately 8 cm of bone growth in the lower limbs.

Surgical treatment and sampling

The orthopedic surgeon first performed medial patellofemoral ligament (MPFL) reconstruction, followed by medial patellar tibial ligament (MPTL) reconstruction and arthroscopic lateral release on the left knee. MPFL reconstruction was performed with the ST folded in two and immobilized at 90˚ of knee flexion. MPTL reconstruction was performed with the medial one-third of the patellar tendon and immobilized at 90˚ of knee flexion through the lower part of the goosefoot. One year after surgery on the left knee surgery, the right knee was treated with MPFL reconstruction and arthroscopic lateral release. In MPFL reconstruction, the same two-fold ST was used for immobilization in the same limb position.

The ST tissue utilized in this study was harvested from the portion that was not needed owing to trimming during graft preparation for MPFL reconstruction. In other words, additional tissue harvesting for this study was not performed for an ethical reason.

Immunostaining and confocal microscopy

To perform tissue immunostaining, 4% paraformaldehyde-fixed, paraffin-embedded tissue sections were stained using a previously described standard protocol [10]. Briefly, sections were deparaffinized and rehydrated in graded alcohols. After antigen retrieval in DAKO Target Retrieval Solution (pH 9; Agilent) for 30 min, the sections were blocked with DAKO Protein Block (serum-free; Agilent) and subjected to immunofluorescence staining at 4 ℃ overnight using the following primary antibodies: goat polyclonal anti-collagen type I (goat IgG; Southern Biotechnology) and mouse monoclonal anti-collagen type III (mouse IgG1; clone FH-7A; Abcam). After washing with 0.1% TritonX-100 in phosphate-buffered saline, the sections were treated with appropriate Alexa-Fluor 488/568-conjugated secondary antibodies (Molecular Probes; Thermo Fisher Scientific) for 1 h at room temperature (approximately 25 ℃) and, thereafter, mounted with 4',6-diamidino-2-phenylindole (DAPI) for nuclear staining. Confocal imaging was performed using an inverted Nikon Eclipse Ti2 confocal microscope (Nikon Instruments/Nikon Corp., Tokyo, Japan) equipped with an Andor Dragonfly spinning disk unit, Andor EMCCD camera (iXon DU888; Andor Technology Ltd., Oxford Instruments), and laser unit (Coherent Inc., Santa Clara, USA). Excitation for DAPI, Alexa-488, and 568 chromophores was provided by 405 nm, 488 nm, and 560 nm lasers, respectively. 

Evaluation and results

**Figure 3:**
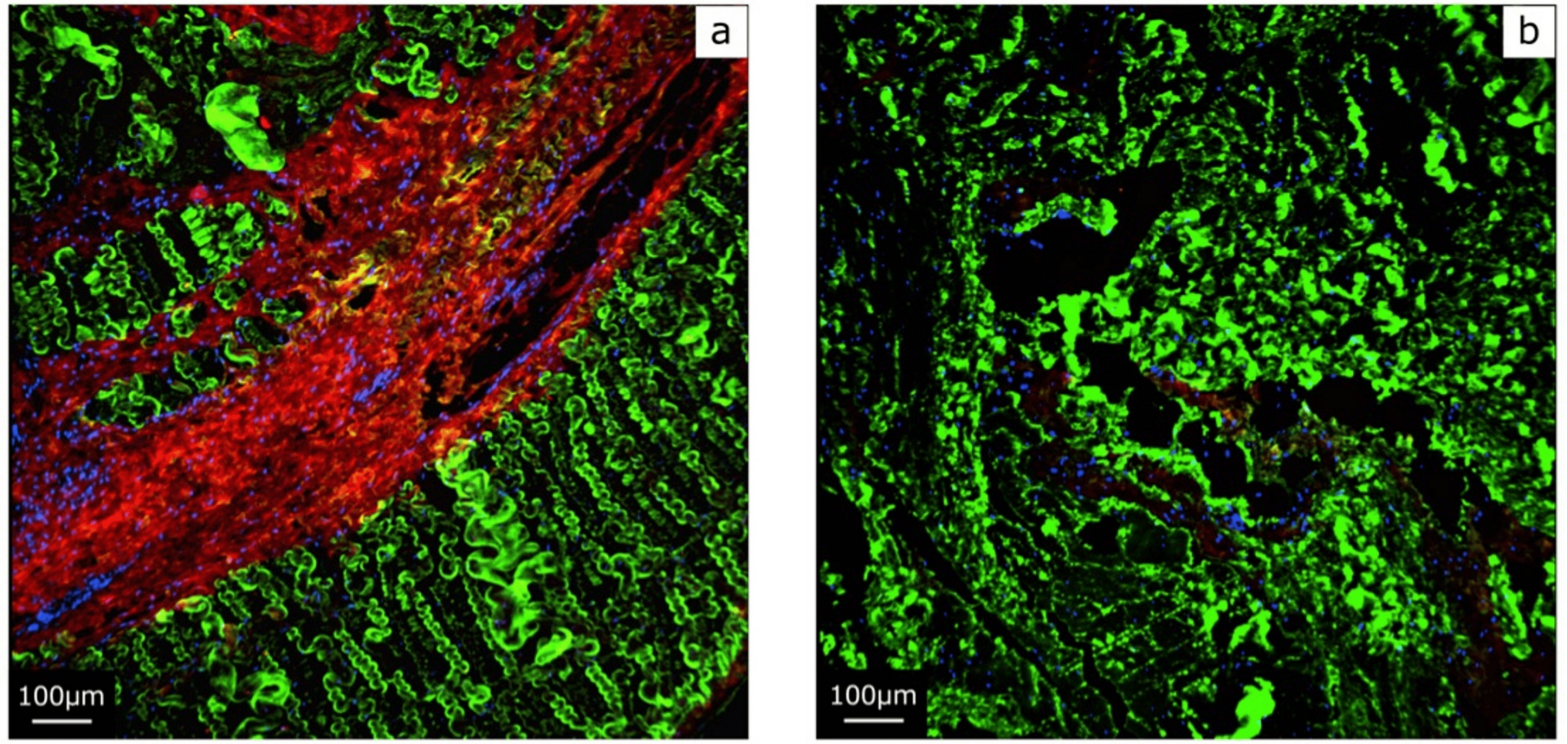
Immunostained images of semitendinosus tendons Type I collagen, type III collagen, and DAPI were colored green, red, and blue, respectively, in semitendinosus tendons collected at 11 (a) and 12 years of age (b). DAPI: 4',6-diamidino-2-phenylindole

The evaluation method was based on previous studies [6]. To quantify type I and type III collagen-positive areas, images of nine sagittal sections were evaluated quantitatively. For quantitative evaluation, we used the Fiji/Image-J (https://fiji.sc). Briefly, images were converted into 8-bit tag image file format (TIFF) files, and the average stained areas for type I and type III collagen-positive regions from three random fields were calculated using the Image-J Analyze Particle tool for each section. Areas of collagen positivity were manually thresholded with a visual comparison with the images to ensure that the tool effectively resolved anti-collagen antibody-stained lesions. Data were expressed as the percentage areas of collagen types per microscopic field. To avoid bias, evaluations were conducted in a two-person blinded fashion. Results showed that the area content of type I collagen in STs collected from the 11-year-old patient was 66% (Figure 3a). A similar study was conducted on STs taken from the same patient one year later, and the area content of type I collagen increased rapidly to 95% (Figure 3b).

## Discussion

This study aimed to determine the physical strength of the ST as an autograft for ACL reconstruction based on its collagen content. An important finding of this study was that the percentage of type I collagen in the ST of an adolescent patient increased with body or bone growth.

The structural stability of collagen, the main component of tendons, is affected by intermolecular cross-linking; particularly in type I collagen, fibers with more cross-linked structures are more tightly bound [11]. In addition, mechanical stress, as typified by traction force, is an essential factor for the maturation of collagen fibers. The relationship between traction force and collagen fibers in tendons has been of interest to researchers; moreover, traction force promotes cross-linking between the amino and carboxy ends of collagen fibers to structurally strengthen the entire fiber [9] and traction force adjusts collagen orientation and increases collagen density [12]. Notably, tendon collagen fibers have a very rapid response to traction force [13].

Recently, the relatively high rate of re-injury in patients with ACL injuries with an open epiphyseal line has become a problem associated with reconstruction using the ST as an autograft [3]. We believe that one of the reasons for this is the microstructural and histological immaturity of the tendon itself in adolescent patients compared to adults [5,6]. Young tendons rupture at lower stresses than mature tendons [14], and this supports our results. The results of this study suggest that the collagen composition of the ST changes dramatically with rapid bone growth in adolescent patients, and its postoperative clinical outcome may be poor when autografted by ST before maturity. However, this study focuses only on physical growth and other factors may have been involved in this dramatic change.

Regarding collagen type and fiber diameter in the skin, which is the same soft tissue as tendons, fibers containing a large amount of type I collagen have a larger diameter; meanwhile, fibers containing a large amount of type III collagen have a smaller diameter [15,16]. In addition, a significant positive correlation between collagen fiber diameter and mechanical strength has been reported [17]. These findings suggest that the mechanical strength of STs in adolescent patients may increase rapidly in a short period (a few months). In addition, these results may provide evidence for the selection of autografts for ACL reconstruction in adolescent patients.

This study had some limitations. First, this study was based on the observation of only one patient's ST; besides, the patient had bilateral patellar dislocation. This is because the collagen composition of tendons and ligaments differs from that of healthy participants [18]. Although this fact may significantly affect the results of this study, it is difficult to frequently collect ST samples from healthy participants from an ethical perspective. Second, the collagen type content of STs resulted in this study was observed immediately after each collection. Consequently, ligamentization in autografts after ACL reconstruction [16] was not considered. This process includes inhibition of necrosis and maturation during the replacement of the grafted tendon with a new ligament [19]. Fragmentation, resorption, neogenesis, and reorganization of collagen fibers of autografts occur in the first few weeks after reconstruction [20]. Therefore, it is possible that the collagen state of the neo-ligamentous tissue was significantly altered compared to that of the autograft immediately after transplantation. However, it is also ethically difficult to evaluate this speculation. Furthermore, the sample used in this study was taken from the edge of the ST, and the collagen type content may differ from that in the parenchyma utilized as autografts for ACL reconstruction. In addition, because no actual mechanical tests were performed, it remains unknown how the strength of the ST essentially changed in response to body or bone growth. To address these limitations, human studies with a higher number of cases and animal studies are warranted.

## Conclusions

The comparison of the two STs obtained from the patient in the first half of their 10^th^ year showed that the type I collagen content of the STs increased rapidly over one year. These results suggest that ST, which is frequently used for ACL reconstruction in adolescent patients, matures rapidly in one year. These results may help to provide a potential basis for the selection of autografts for ACL reconstruction in adolescent patients.
